# Cooperation without Culture? The Null Effect of Generalized Trust on Intentional Homicide: A Cross-National Panel Analysis, 1995–2009

**DOI:** 10.1371/journal.pone.0059511

**Published:** 2013-03-19

**Authors:** Blaine Robbins

**Affiliations:** Department of Sociology, University of Washington, Seattle, Washington, United States of America; George Mason University/Krasnow Institute for Advanced Study, United States of America

## Abstract

Sociologists, political scientists, and economists all suggest that culture plays a pivotal role in the development of large-scale cooperation. In this study, I used generalized trust as a measure of culture to explore if and how culture impacts intentional homicide, my operationalization of cooperation. I compiled multiple cross-national data sets and used pooled time-series linear regression, single-equation instrumental-variables linear regression, and fixed- and random-effects estimation techniques on an unbalanced panel of 118 countries and 232 observations spread over a 15-year time period. Results suggest that culture and large-scale cooperation form a tenuous relationship, while economic factors such as development, inequality, and geopolitics appear to drive large-scale cooperation.

## Introduction

Maintaining large-scale cooperation over long periods of time is a difficult and arduous task for any society. Conventional models of social order, based on the assumption of self-regarding individuals, predict zero cooperation and rampant social conflict in the absence of external authorities [1]. Yet, recent theory and findings suggest that cultural factors, such as generalized morality or norms of reciprocity, in addition to structural features of a society like economic development, urbanization, and ethnic fractionalization may play an important part in promoting large-scale cooperation among groups and societies with or without government control [2], [3], [4], [5], [6], [7], [8].

Much of the evidence in support of culture is quite compelling and largely comes from laboratory experiments showing that culture promotes cooperation [9], [10]. However, unless the relation between culture and large-scale cooperation is investigated in concrete real-life settings, where one can account for context-specific structural factors, the ultimate impact of culture on social order is difficult to evaluate. Although previous studies have explored the relationship between culture and large-scale cooperation with diverse national and cross-national populations [11], [12], [13] and have documented the importance of some cultural elements [14], [15], [16], [17], reliable evidence on the extent to which variation in measures of culture, such as generalized trust, affects real-life measures of large-scale cooperation, such as intentional homicide, through time is altogether missing [18].

I combined cross-national data on intentional homicide with an observational measure of culture – generalized trust – to form an unbalanced 15-year panel composed of 118 countries and 232 observations. With this data, I investigated the relationship between generalized trust and intentional homicide using pooled time-series linear regression, single-equation instrumental-variables linear regression, fixed- and random-effects linear panel models, and instrumental-variables two-stage least squares random-effects linear panel models. I also explored whether the culture-cooperation relationship was conditional on social structure, be it urbanization, economic development, or political institutions. In doing so, I aimed to underscore the robustness of my findings: that large-scale cooperation, measured as intentional homicide, stems not from cultural factors like generalized trust, but from economic features of a society, namely economic development, economic equality, and geopolitics. In all, I produced the first study to investigate the impact of culture on large-scale cooperation through time.

## Methods

### Ethics Statement

All data used for the present study is secondary and publicly available. Human subjects were not directly contacted or surveyed by the author. The study was approved by the Human Subjects Division of the author’s university.

### Measures

To measure large-scale cooperation I used an operationalization of intentional homicide drawn from the most recent data published by the United Nations Office on Drugs and Crime (UNODC) in their 2011 report “Global Study on Homicide.” The document pools indicators of intentional homicide from the World Health Organization (WHO), the United Nations Surveys on Crime Trends and the Operations of Criminal Justice Systems (UN-CTS), and national police statistics. I also supplemented missing UNODC data with World Bank indicators of intentional homicide. I natural logged all intentional homicide data.

To measure culture, I used an operationalization of generalized trust drawn from various cross-national public-opinion data sets, including the Afro Barometer, the Arab Barometer, the Asian Barometer, the European Values Study (EVS), the Latino Barometer, and the World Values Survey (WVS). With this data, I followed the WVS wave structure and compiled a three-wave unbalanced panel spanning 15 years (1995–1998, 1999–2004, 2005–2009). I then aggregated generalized trust responses to create a measure of the proportion of respondents – multiplied by 100– who said that most people can be trusted (ranging from 0 to 100) when asked the following question: “Generally speaking, would you say that most people can be trusted or that you need to be very careful in dealing with people?” This is the prevailing measure of generalized trust used in the social sciences [19], [20], [21], [22], [23], [24], [25], [26], [27]. All generalized trust data were frequency weighted when available (e.g., WVS S017).

To account for possible confounding effects of social structure, I included a number of time-varying indicators in my models common to political science, sociology, and homicide studies [18], [28]: (1) the property rights indicator from the Heritage Foundation’s Index of Economic Freedom was used to operationalize political institutions that allow for the accumulation and security of private property; (2) the natural log of gross domestic product (GDP) per capita (constant year 2000 US$) from the World Bank was used to capture economic development and modernization; (3) the natural log of the gini coefficient from the World Bank, the OECD, and the CIA World Factbook was used to measure a country’s income inequality; (4) an index from the Encyclopedia Britannica and the CIA World Factbook, which consists of the percent largest ethnic and linguistic groups in a country summed and divided by two (α = .78), was used to capture ethno-linguistic homogeneity; and, (5) the percent of the population residing in urban areas from the World Bank was used to measure urbanization. I also controlled for a number of time-invariant dummy variables. These included measures for Latin American countries, African countries, and former communist countries.

Finally, I used three time-invariant instrumental variables common to the generalized trust literature for my generalized method of moments (GMM) single-equation instrumental-variables regression and generalized two-stage least squares (G2SLS) random-effects linear panel analysis [5], [19], [20], [25], [29], [30]. These included measures for (a) countries with absolute or constitutional monarchies (i.e., Monarchy), (b) countries with Scandinavian cultural heritages such as Denmark, Finland, Iceland, Norway, and Sweden (i.e., Nordic), and (c) a country’s average low temperature in the coldest month of the year in Fahrenheit scale (i.e., Temperature) from the website Weatherbase (www.weatherbase.com). See the author’s website (www.blainerobbins.com) for all data used in the present analysis by country, wave, and survey year, and refer to Table 1 for descriptive statistics.

**Table 1:** Description of variables and summary statistics.

Variables	Unit	Country N	Mean	SD	Min	Max
Generalized trust	Proportion of sample who believe that others can be trusted.	118	26.28	14.35	3.35	76.12
Property rights	10 = Property protection to 1 = no property protection.	118	54.33	23.19	10	95
ln(GDP)	ln(gross domestic product per capita, constant year 2000 US$).	118	8.16	1.46	4.89	10.91
ln(gini)	ln(absolute inequality from 0–100).	117	3.61	.26	2.82	4.31
Urbanization	Urban population (% total population).	118	61.67	20.06	12.54	100
Ethnolinguistic homogeneity	(% largest ethnic group+% largest linguistic group)/2.	118	75.25	18.59	10.52	99.86
Latin America	1 = Caribbean, Central and South American country, 0 = otherwise.	118	.20	–	0	1
Africa	1 = African country, 0 = otherwise.	118	.12	–	0	1
Former communist	1 = former Marxist-Leninist state, 0 = otherwise.	118	.30	–	0	1
Monarchy	1 = Monarchy, 0 = otherwise.	118	.18	–	0	1
Nordic	1 = Denmark, Finland, Iceland, Norway, and Sweden, 0 = otherwise.	118	.05	–	0	1
Temperature	Average low temperature (Fahrenheit) in the coldest month of the year.	114	40.19	20.46	−15.9	74.9

### Model Specification

In my first set of estimates, I modeled the natural log of intentional homicide as a function of generalized trust and control variables by pooling the time-series of the country sample and using ordinary least-squares (OLS) linear regression. I then used the same pooled time-series country sample and explored the impact of generalized trust on homicide with GMM single-equation instrumental-variables linear regression. Then, I used fixed- and random-effects linear panel models as well G2SLS random-effects linear panel models [31] to control for unobserved heterogeneity between countries and to investigate the exogeneity of generalized trust. Finally, I explored conditional relationships between generalized trust and (a) urbanization, (b) economic development, and (c) political-institutional dependence with pooled time-series OLS linear regression as well as random-effects linear panel models. All models were run using Stata 12.1.

## Results

For all models, I included every observation as none of the observations produced both large residuals and high leverage (i.e., influence) – DfBetas and tests of Cook’s distance yielded reasonable values as well; I included a squared term for property rights; I found that multicollinearity was only an issue for the property rights polynomial and that centering the two property rights terms did not substantively alter the results presented here – the variance inflation factors (i.e., VIF) for all other coefficients in the pooled-time series OLS models were well below (less than 5.5) the typical cut-off value of 10.0 [32]; and, I provided two-tailed tests throughout.

### Pooled Time-Series OLS and GMM Analysis

**Figure 1:**
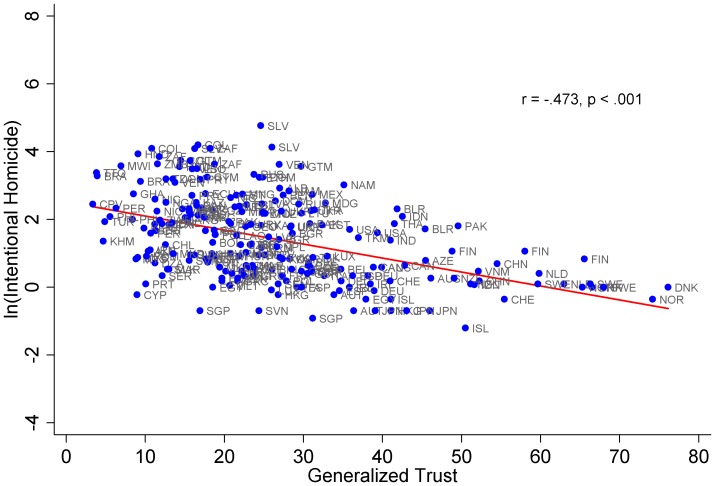
Generalized trust and ln(intentional homicide), 1995 to 2009.

Table 2 presents a series of nested pooled time-series OLS regression and GMM single-equation instrumental-variables linear regression models. All models in Table 2 include wave dummies and cluster-robust standard errors by country. Model 1 includes three classic predictors of cooperation (or intentional homicide): culture, politics, and economy operationalized as generalized trust, property rights, and natural log of gross domestic product, respectively. Model 1 indicates that all variables have the expected signs and are statistically significant. As anticipated (see Figure 1), increases in generalized trust reduce intentional homicide, which supports prior research [11], [16], [33], [34]. Model 1 also shows that increases in property rights promote intentional homicide at low levels of property rights protection. This positive effect, however, attenuates as property rights increase. In other words, property rights institutions increase intentional homicide in countries that have low levels of property rights protection but reduce intentional homicide for countries that have fairly robust property rights institutions. The results suggest that regimes with low and high levels of property rights protection yield the lowest rates of intentional homicide, while countries with intermediate levels generate the highest rates of intentional homicide (see the following for similar arguments: [35], [36]). Finally, model 1 reveals intentional homicide to be negatively related to natural log GDP. Overall, the terms in model 1 do an excellent job of accounting for variance in intentional homicide (R^2^ = .41) and tend to parallel prior results [28].

**Table 2:** Intentional homicide and generalized trust: A pooled time-series analysis.

Estimation method	OLS	OLS	OLS	OLS	OLS	GMM
Parameters	Model 1^a^	Model 2^a^	Model 3^a^	Model 4^b^	Model 5^b^	Model 6c,d
Generalized trust	−.019**	−.005	.0007	−.016*	.002	.008
	(.005)	(.006)	(.005)	(.008)	(.006)	(.010)
Property rights	.04*	.036*	.029*	.065**	.037*	.033*
	(.016)	(.014)	(.014)	(.019)	(.019)	(.015)
Property rights^2^	−.0004**	−.0004*	−.0002†	−.0006**	−.0003	−.0003*
	(.0001)	(.0001)	(.0001)	(.0002)	(.0002)	(.0001)
ln(GDP)	−.316***	−.264**	−.247*	−.358**	−.249†	−.259*
	(.085)	(.074)	(.110)	(.119)	(.147)	(.108)
ln(gini)		2.046***	1.089*		1.173*	1.191*
		(.348)	(.473)		(.527)	(.476)
Urbanization			−.003		−.004	−.002
			(.006)		(.007)	(.005)
Ethnolinguistic homogeneity			−.008†		−.008	−.008†
			(.004)		(.007)	(.004)
Latin America			1.357***		1.069**	1.393***
			(.300)		(.362)	(.302)
Africa			.437		.551	.385
			(.338)		(.475)	(.328)
Former communist			.510**		.341	.569**
			(.183)		(.236)	(.187)
Wave 2	−.247*	−.301**	−.204†			−.169
	(.122)	(.114)	(.113)			(.11)
Wave 3	−.279*	−.384**	−.277*	−.391***	−.306**	−.249*
	(.124)	(.118)	(.128)	(.100)	(.094)	(.126)
Constant	3.92***	−4.11**	−.711	3.581***	−1.11	−1.357
	(.602)	(1.469)	(2.019)	(.769)	(2.276)	(2.092)
No. observations	232	231	230	133	130	225
No. countries	118	117	117	86	85	113
R^2^	.411	.554	.637	.423	.650	.632
RMSE	.965	.841	.768	.969	.776	.746
First Stage Partial R^2^	–	–	–	–	–	.256
First Stage F-Statistic	–	–	–	–	–	22.619
Hansen's J-Statistic	–	–	–	–	–	.517

*** p<.001,

** p<.01,

* p<.05,

† p<.10 (cluster-robust standard errors in parentheses).

a Linear regression with contemporaneous independent variables and wave dummies.

b Linear regression with lagged t-1 independent variables and wave dummy.

c GMM instrumental-variables regression with contemporaneous independent variables and wave dummies.

d Instruments are dummies for Nordic culture and monarchies, and the average low temperature in the coldest month of the year.

Models 2 and 3 include nested controls for economic inequality (i.e., natural log of the gini coefficient), urbanization (i.e., % total urban population), social cleavages (i.e., ethnolinguistic homogeneity), and regional dummies (i.e., Latin American, African, and former communist countries). Model 2 shows generalized trust and intentional homicide to be statistically unrelated once I control for income inequality. This suggests that the relationship between cooperation and culture is either confounded with or mediated by income inequality. In either case, this model calls into question the direct effect of culture on cooperation. Note that all other terms in model 2 parallel those found in model 1 and that the R^2^ increased substantially from. 41 to.55. Finally, by including the remaining control variables, model 3 shows that culture remains unrelated to cooperation and that the parameter estimates and standard errors of the control variables parallel prior work. Model 3 also accounts for approximately 64% of the variance in intentional homicide.

In models 4 and 5 I follow the same procedures found in models 2 and 3, respectively, but include lagged t-1 independent variables instead of contemporaneous independent variables. The results are similar: once I control for income inequality, the statistically significant effect of generalized trust on intentional homicide dissolves. Finally, model 6 presents a GMM single-equation instrumental-variables linear regression model and uses Nordic, Monarchy, and Temperature as instruments. First, results suggest that the three instruments are not weak: the first stage partial-R^2^ is greater than 0.25 and the Cragg-Donald first-stage F-statistic is above the typical cut-off value of 10 [37], [38]. Second, the Hansen’s J-statistic is above the 5% test level, which means that all three instruments are valid and that the structural model is specified correctly. These instrument tests confirm previous results [19], [25], [30]. Overall, the findings in model 6 are comparable, although not equivalent, to model 3 and suggest that regardless of instruments, culture does not directly produce cooperation.

In short, the pooled time-series OLS and GMM single-equation instrumental-variables analysis reveals the following: property rights and intentional homicide form a curvilinear relationship – intentional homicide increases at an decreasing rate with property rights protection; economic growth (i.e., GDP) undermines intentional homicide; income inequality fosters intentional homicide; Latin American countries as well as former communist countries are generally less cooperative, on average, than other countries; and culture – or generalized trust – does not produce cooperation (i.e., intentional homicide).

### Fixed- and Random-Effects Panel Analysis

To control for unobservable time-invariant factors and to explore how changes in predictors over time affect cooperation, I estimated a series of fixed- and random-effects panel models. Fixed-effects models estimate differences within countries while random-effects models estimate differences across countries as well as across time-periods. To test whether the variation across countries is correlated with the predictors in the models (i.e., independence assumption), I used the Hausman specification test [39]; the test indicates that fixed-effects estimation techniques should be used (the test statistic for models 3 and 4 in Table 3 is χ^2^ (8) = 21.34, which rejects the null hypothesis of independence). Although fixed-effects estimators are inefficient in unbalanced datasets with a small number observations per unit and approximately time-invariant treatments (e.g., generalized trust) [40], recent work suggests using fixed-effects over random-effects under such conditions despite issues of possible inefficiency [41]. Nevertheless, the reader should be aware of possible inefficiency in the fixed-effects estimator for the present study.

**Table 3:** Intentional homicide and generalized trust: A fixed- and random-effects panel analysis.

Estimation method	RE	FE	RE	FE	RE	FE	RE	RE
Parameters	Model 1^a^	Model 2^b^	Model 3^a^	Model 4^b^	Model 5^c^	Model 6^d^	Model 7e,g	Model 8f,g
Generalized trust	−.012**	−.005	−.006	−.002	−.0008	.002	.002	.011
	(.004)	(.005)	(.004)	(.005)	(.006)	(.009)	(.011)	(.014)
Property rights	.011	−.009	.008	−.005	.029†	.049	.016	.037†
	(.011)	(.013)	(.011)	(.014)	(.018)	(.044)	(.012)	(.02)
Property rights^2^	−.0001	.0001	−.0001	.00005	−.0002	−.0005	−.0001	−.0003
	(.0001)	(.0001)	(.0001)	(.0001)	(.0002)	(.0004)	(.0001)	(.0002)
ln(GDP)	−.394***	−.72**	−.31***	−.608**	−.305*	−1.823*	−.282**	−.304*
	(.064)	(.213)	(.088)	(.222)	(.130)	(.712)	(.086)	(.127)
ln(gini)			−.0002	−1.221*	.857†	.974	.32	1.233*
			(.322)	(.491)	(.45)	(1.019)	(.33)	(.486)
Urbanization			.0007	.015	−.002	.085*	−.0006	−.0009
			(.005)	(.02)	(.007)	(.038)	(.005)	(.007)
Ethnolinguistic homogeneity			−.008*	−.009	−.007	.022	−.009*	−.007
			(.004)	(.008)	(.005)	(.015)	(.004)	(.005)
Latin America			1.675***		1.182***		1.633***	1.161***
			(.261)		(.319)		(.254)	(.321)
Africa			.609*		.481		.545*	.461
			(.256)		(.347)		(.24)	(.326)
Former communist			.293		.267		.422*	.422
			(.198)		(.269)		(.181)	(.276)
Wave 2	−.078	−.026	−.083	−.039			−.079	
	(.069)	(.072)	(.069)	(.075)			(.082)	
Wave 3	−.106	−.011	−.148*	−.055	−.196**	−.123	−.165†	−.188*
	(.073)	(.104)	(.076)	(.119)	(.064)	(.091)	(.085)	(.078)
Constant	4.738***	7.721***	4.034**	10.758***	.422	4.725	2.391	−1.480
	(.549)	(1.764)	(1.394)	(2.887)	(1.96)	(5.3)	(1.465)	(2.225)
No. observations	232	232	230	230	130	130	225	129
No. countries	118	118	117	117	85	85	113	84
R^2^ within	.189	.231	.206	.285	.192	.393	.150	.137
R^2^ between	.398	.327	.611	.144	.667	.329	.620	.666
σ_ν_	.882	1.098	.711	1.149	.709	1.501	.687	.710
σ_e_	.307	.307	.302	.302	.282	.282	.405	.377
ρ	.892	.927	.847	.936	.863	.966	.741	.780

*** p<.001,

** p<.01,

* p<.05,

† p<.10 (standard errors in parentheses).

a Random-effects regression with contemporaneous independent variables and wave dummies.

b Fixed-effects regression with contemporaneous independent variables and wave dummies.

c Random-effects regression with lagged t-1 independent variables and wave dummy.

d Fixed-effects regression with lagged t-1 independent variables and wave dummy.

e G2SLS random-effects regression with contemporaneous independent variables and wave dummies.

f G2SLS random-effects regression with lagged t-1 independent variables and wave dummy.

g Instruments are dummies for Nordic culture and monarchies, and the average low temperature in the coldest month of the year.

Table 3, like Table 2, presents a series of nested models but, instead, uses fixed- and random-effects as well as G2SLS random-effects estimation techniques. With the exception of property rights, income inequality, and former communist countries, results suggest that the random-effects models (i.e., models 1 and 3) parallel the pooled time-series OLS regression models found in Table 2. In other words, generalized trust remains statistically unrelated to intentional homicide even when I treat differences within- and between-countries across time as random variables, and when I loosen the assumption of no unique attributes of countries and no universal effects across time. Interestingly, with the exception of model 2, the statistical significance of generalized trust for models using a fixed-effects estimator is comparable to models using a random-effects estimator, regardless of contemporaneous or lagged t-1 independent variables. Finally, in models 7 and 8 I use a G2SLS random-effects estimator with contemporaneous and lagged t-1 independent variables, respectively. Once again, I find generalized trust to be statistically unrelated to intentional homicide. Although not shown, results for generalized trust were similar to those presented in Table 3 when run with two- and three-wave balanced panel models or when using population-average estimators. Finally, the fixed-effects estimator found in models 2 and 6 yielded statistically insignificant results for generalized trust even in the absence of covariates and controls.

In short, the fixed- and random-effects linear panel models as well as the G2SLS random-effects linear panel models presented in Table 3 suggest the following: first, regardless of the estimator, GDP and Latin American countries statistically decrease and increase intentional homicide, respectively; second, generalized trust is statistically unrelated to intentional homicide once I either control for income inequality or employ fixed-effects estimation techniques.

### Conditional Analysis

The fixed- and random-effects estimates appear to simultaneously support and challenge prior work using OLS or 2SLS cross-sectional designs [11], [12], [16]. Since this is the case, I investigated classic interaction effects and conditional propositions. For all three interaction effects, I expect generalized trust to decrease intentional homicide. But I also expect this negative effect to attenuate and weaken as urbanization increases [42]; as economic development and modernization increases [43]; or, as political-institutional dependence increases [44], [45]. In other words, I expect urbanization, modernization, or political-institutional dependence to undermine the negative effect of generalized trust on intentional homicide. The models in Table 4, however, reveal little support for these conditional hypotheses. Although the signs of the coefficients are in the expected direction, none of the higher-order terms (i.e., interaction effects) in the OLS or random-effects models are statistically significant at the 0.05 level. Moreover, the online supporting material (Figure S1, Figure S2, and Figure S3) shows that the marginal effects of generalized trust on intentional homicide as either property rights, ln(GDP), or urbanization increases (see models 4–6, Table 4) are statistically insignificant [46]. While not presented, the null effects for the conditional relationships were also found with fixed-effects linear panel models, and the interaction effect between generalized trust and property rights-squared was statistically insignificant.

**Table 4:** Intentional homicide and generalized trust: A pooled time-series conditional analysis.

Estimation method	OLS	OLS	OLS	RE	RE	RE
Parameters	Model 1^a^	Model 2^a^	Model 3^a^	Model 4^b^	Model 5^b^	Model 6^b^
Generalized trust	−.013	−.041†	−.023†	−.01	−.023	−.018
	(.011)	(.023)	(.014)	(.009)	(.021)	(.011)
Property rights	−.0008	.031*	.03*	−.0009	.009	.009
	(.007)	(.013)	(.013)	(.005)	(.011)	(.011)
Property rights^2^		−.0003*	−.0003†		−.0001	−.0001
		(.0001)	(.0001)		(.0001)	(.0001)
ln(GDP)	−.267*	−.364**	−.249*	−.318***	−.36**	−.317***
	(.111)	(.124)	(.108)	(.088)	(.108)	(.088)
ln(gini)	1.145*	1.169*	1.205*	.008	.026	.035
	(.491)	(.48)	(.498)	(.321)	(.322)	(.321)
Urbanization	−.004	−.003	−.012†	.0005	.0006	−.004
	(.006)	(.006)	(.007)	(.0005)	(.005)	(.006)
Ethnolinguistic homogeneity	−.008†	−.008†	−.008†	−.008*	−.008*	−.008*
	(.004)	(.004)	(.004)	(.004)	(.004)	(.004)
Latin America	1.391***	1.316***	1.315***	1.698***	1.673***	1.667***
	(.307)	(.301)	(.303)	(.256)	(.255)	(.254)
Africa	.428	.356	.356	.606*	.576*	.562*
	(.352)	(.346)	(.343)	(.258)	(.258)	(.257)
Former communist	.571**	.531**	.514**	.311	.307	.30
	(.18)	(.184)	(.182)	(.198)	(.197)	(.196)
Property × trust	.0002			.00007		
	(.0002)			(.0001)		
ln(GDP) × trust		.005†			.002	
		(.003)			(.003)	
Urban × trust			.0004†			.0002
			(.0002)			(.0002)
Wave 2	−.228†	−.189†	−.191†	−.089	−.084	−.08
	(.116)	(.111)	(.111)	(.068)	(.069)	(.069)
Wave 3	−.331*	−.279*	−.287*	−.163*	−.159*	−.157*
	(.141)	(.129)	(.128)	(.076)	(.078)	(.076)
Constant	.177	−.014	−.534	4.352**	4.364**	4.258**
	(2.098)	(2.089)	(2.053)	(1.403)	(1.479)	(1.416)
No. observations	230	230	230	230	230	230
No. countries	117	117	117	117	117	117
R^2^ within	–	–	–	.211	.198	.20
R^2^ between	–	–	–	.608	.617	.619
σ_ν_	–	–	–	.717	.703	.701
σ_e_	–	–	–	.302	.303	.303
ρ	–	–	–	.859	.843	.843

*** p<.001

** p<.01

* p<.05

† p<.10 (OLS = cluster-robust standard errors; RE = standard errors)

a Linear regression with contemporaneous independent variables and wave dummies

b Random-effects regression with contemporaneous independent variables and wave dummies

### Sensitivity Analysis

To explore model sensitivity, I re-estimated the models in Tables 2–4 with alternative measures of income inequality from Solt’s Standardized World Income Inequality Database, v.3.1 [47], which includes comparable measures across time and countries for net and gross income inequality. While my income inequality measure was highly correlated with Solt’s net income inequality measure (*r* = .95), this was not the case for Solt’s gross income inequality measure (*r* = .66). In spite of this variation, substituting my income inequality measure for either Solt’s net or gross income inequality measure resulted in only minor substantive differences. This was especially the case for (a) the direct effect of generalized trust on intentional homicide in model 2, Table 2 when controlling for gross income inequality (β = −0.012, *SE* = 0. 006, *p*<.05) but not when controlling for net income inequality (β = 0.002, *SE* = 0. 006, *p* = .69), (b) the interaction effect between generalized trust and GDP in model 2, Table 4 when controlling for net income inequality (β = 0.006, *SE* = 0. 003, *p*<.05) but not when controlling for gross income inequality (β = 0.003, *SE* = 0. 003, *p* = .31), and (c) the interaction effect between generalized trust and urbanization in model 3, Table 4 when controlling for net income inequality (β = 0.0004, *SE* = 0. 0002, *p*<.05) but not when controlling for gross income inequality (β = 0.0002, *SE* = 0. 0002, *p* = .21). Note: only the OLS estimator yielded substantive differences in coefficients and standard errors, which are biased and inconsistent due to the time-series nature of the data. All other estimators (e.g., RE, FE, 2SLS, etc.) produced substantively similar results regardless of the income inequality measure used.

Finally, I explored a number of alternative model specifications for time-series, cross-sectional data analysis [48], which included AR(1) models, DL(1) models, LDV models, and ARDL(1, 1) models with random- and fixed-effects estimators where applicable (AR: autoregressive; DL: distributed lag; LDV: lagged dependent variable; ARDL: autoregressive, distributed lag). Regardless of the estimator, the direct, conditional, or marginal effect of generalized trust on intentional homicide was statistically insignificant (results available upon request).

## Discussion

My findings establish that some cultural elements of a society are unimportant for large-scale cooperation. The results do not identify complementarities between observational measures of large-scale cooperation – intentional homicide – and survey measures of culture (i.e., generalized trust), which, taken together, also fails to provide external validity to laboratory experiments [49], [50]. My study does, however, contribute to accumulating evidence suggesting that effective large-scale cooperation is not necessarily the direct result of cultural attributes like generalized morality but a consequence of economic development, economic inequality, and geopolitics [18], [28].

While my results question the causal relationship between culture and cooperation, my models do not entirely discount the effect of culture. When considering recent research [51], [52], the finding that cooperation varies in response to economic factors might suggest that the effect of generalized trust on intentional homicide is either partially mediated by economic development or fully mediated by economic inequality. In either case, culture might indirectly affect large-scale cooperation through one or both of these economic factors. Moreover, my geopolitical measures of Latin America, Africa, and former communist regimes undoubtedly capture elements of culture that may account for higher rates of intentional homicide in these regions [53], [54]. The content of these unique cultural elements and how they produce large-scale cooperation is a task for future research. In spite of this, my results provide a clear message: culture, measured as generalized trust, does not directly, or even conditionally, beget large-scale cooperation.

## Supporting Information

Figure S1 **Marginal effect of generalized trust on intentional homicide as property rights increases.**
(DOC)

Figure S2 **Marginal effect of generalized trust on intentional homicide as ln(GDP) increases.**
(DOC)

Figure S3 **Marginal effect of generalized trust on intentional homicide as urbanization increases.**
(DOC)
