# Supplementary material for: Cooperation without Culture? The Null Effect of Generalized Trust on Intentional Homicide: A Cross-National Panel Analysis, 1995–2009
Source: PLoS One. 2013 Mar 19;8(3):e59511. doi: 10.1371/journal.pone.0059511 (PMC3602298; doi:10.1371/journal.pone.0059511)
Supplement: Figure S2 — Marginal effect of generalized trust on intentional homicide as ln(GDP) increases. (DOC) [file pone.0059511.s002.doc]

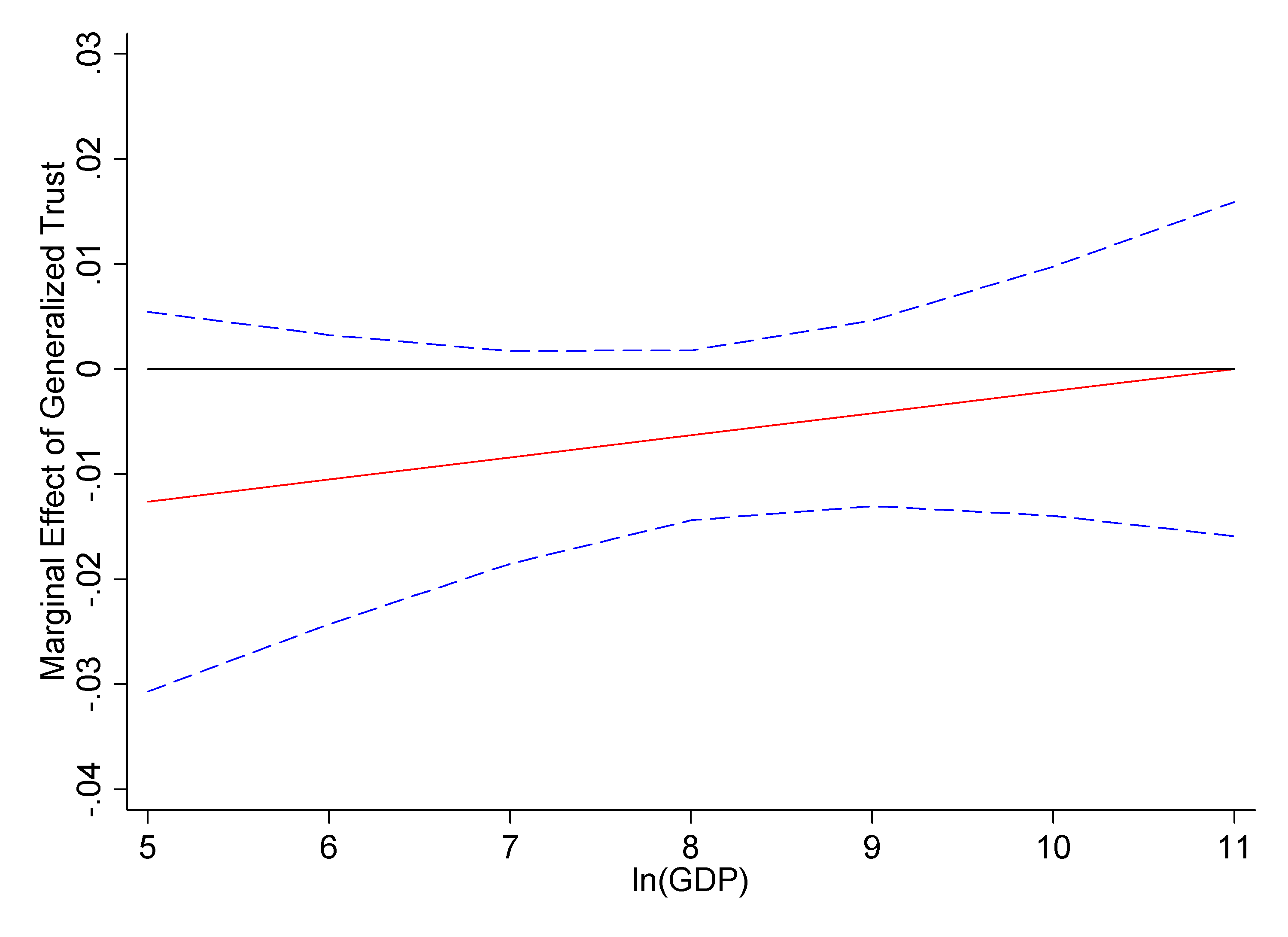
*Note*: marginal effect of generalized trust on intentional homicide as ln(GDP) increases (95% confidence interval in blue, marginal effect line in red, zero line in black). Note that at no point are both the upper and lower bounds of the confidence interval above or below the zero line in black, which suggests a statistically insignificant marginal effect of generalized trust on intentional homicide as ln(GDP) increases. Estimates derived from model 5, Table 4.
